# Who Pulled the Bell-rope?

**Published:** 1891-01

**Authors:** 


### WHO PULLED THE BELL-ROPE?

The author of the following statement had been an engineer with a big reputation as a "runner" in the years gone by, but, on account of failing nerves and eyesight, was now enjoying an easy berth around the shops, says the Kansas City "Star."

It was when the old Y. M. & B. was first opened up, he began. I was pulling passenger, and took the first coach over the road. I got a good run, all day work, and was holdin' her down as a good thing. 'Bout a year after we'd got to doin' a good business I had some extra runnin' and lost my turn for a while, and run nights all of the time. It was my last trip before I'd get back to my own run, and I was feelin' glad to get on to the day "trick" again. We'd had some mighty bad weather, and lots of water fell. Our track was in pretty good shape, though, and we didn't much fear washouts, so we kept up with the "card" pretty well. On the night I spoke about I was on No. 2. We had a heavy train, but the machine I had was able to "get there," and I was on time till we struck a freight that couldn't take the siding. They "swung us down," and we side-tracked until the freight got away. I was pretty warm over losing the time, and when we lit out of there I pulled her right up to the notch, and she went for all she was worth. We were makin' about 45 miles an hour, and when we reached the "fill" east of Wildcat, I worked steam all the way down. We were 'bout half-way to the creek when the bell rang. I worked mighty quick, but it was down hill, and the rails were wet, and I didn't get stopped until the pilot was almost over the bridge—or where the bridge ought to be—'cause when I stopped the head-light was shining over a chasm. The bridge was washed away. Gad! You can tell just 'bout how I felt. My fireman nearly fainted, and I wasn't far behind him. Well, after we stopped, the conductor, a smart chap, with a fancy lamp and rubber collar, came a runnin' up wantin' to know why I stopped.

"'Cause the bell rang. What did you pull the rope for?' I says.

"I didn't," says he.

"Well, who did?" I says.

"No one," says he, hot like.

"Well, some one pulled it or I wouldn't a stopped," says I.

The conductor looked at me a minute, and just then the brakeman came up.

"Did you pull the rope, Joe,?" said the "con."

"No," says Joe.

Just of a sudden a thought struck me, and I told the "braky" to ask the porter. The "coon" hadn't pulled the bell, and the passengers in his car were all asleep until I jerked them endways with the "air." I took the conductor around to the front end and showed him the bridge. He was scared to death, and we went back together through the train to see who pulled the bell-rope, but every mother's son of them swore it wasn't touched. I began to get scared again, and told them about the bridge, and every body came out to look at it. We couldn't find any body who gave the signal, and after we'd flagged back to the station I got to thinkin' more and more, and I came to the opinion that the bell was rung by Providence. There was 150 people on the train, and if that bell hadn't rung I'd a took them all over into the Wildcat, and dropped them about 100 into the water. There wouldn't been any body left to tell about it either.

The superintendent looked into the thing after I reported, and had me and Joe up "on the carpet" twice, but we both heard the bell and swore to it. Some chap got out a long explanation that the bell-rope was tight stretched, and we struck a low joint coming down the hill, when one end of the coach sagged, and, the rope being tight, it rung the bell, but I don't believe it. *It was Providence that did it, and I know it*, and *I've never swore on oath since, and never will.*

